# A case of Lyme disease complicated by the Jarisch-Herxheimer reaction and coinfection with Babesia

**DOI:** 10.1016/j.jdcr.2022.11.023

**Published:** 2022-12-01

**Authors:** Maria Karim, Allen N. Sapadin

**Affiliations:** a Hackensack Meridian School of Medicine, Nutley, New Jersey; b Department of Dermatology, Icahn School of Medicine at Mount Sinai, New York, New York

**Keywords:** babesiosis coinfection, Jarisch-Herxheimer reaction, Lyme disease, JHR, Jarisch-Herxheimer reaction

## Introduction

Lyme disease is a prevalent zoonosis transmitted by the spirochete *Borrelia burgdorferi*. Acute Lyme disease is characterized by the hallmark rash of erythema migrans at the site of the tick bite, which is often the diagnostic clue. The Jarisch-Herxheimer reaction (JHR) is a transient clinical phenomenon that develops within 24 hours after the initiation of antibiotic therapy for spirochetal infections, rarely reported in association with Lyme disease.^1^ Lyme disease is the most frequently transmitted infection by the *Ixodes* tick, although other pathogens may be simultaneously transmitted.^2^ Patients coinfected with multiple tick-borne illnesses demonstrate nonspecific symptoms and follow an unpredictable disease course, presenting a diagnostic challenge. Herein, the authors report a case of Lyme disease complicated by the JHR and coinfection with Babesia occurring in an endemic area.

## Case Report

A healthy 68-year-old man presented for evaluation of an acute non-pruritic rash on his abdomen. The rash was initially noticed 3 days before, after returning from a trip to Connecticut. Over the last 3 days, the lesion rapidly enlarged and deepened in color. The patient denied having any fever, chills, nausea, vomiting, headache, myalgias, or joint pain. He denied a history of a recent tick bite, although activities on his trip included gardening in a wooded area.

**Fig 1:**
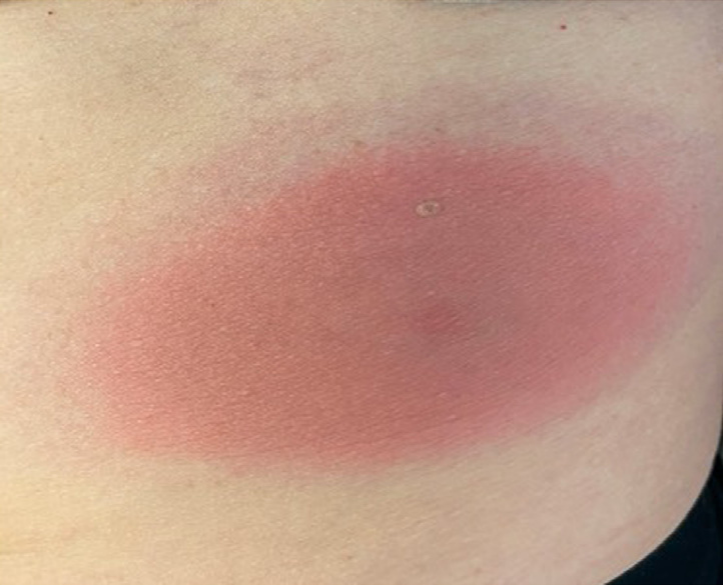
Erythema migrans in a patient with Lyme disease at initial presentation. An 11-cm red, annular plaque with defined borders and central clearing on the left side of the lateral abdomen.

Physical examination revealed an 11-cm red, annular plaque with defined borders and central clearing on the left side of the lateral abdomen and a central tick bite. This was warmer to the touch than the surrounding skin (Fig 1). Differential diagnoses included tinea corporis and erythema annulare centrifugum. Laboratory evaluation revealed negative Lyme IgG and immunoglobulin M antibodies. A 6-mm punch biopsy sample revealed changes consistent with spongiotic dermatitis, acanthosis and intercellular edema in the epidermis, and a perivascular mononuclear infiltrate in the dermis. Because of the patient’s clinical history, classic appearance of the lesion, and biopsy findings, suspicion of Lyme disease was high and the patient was empirically treated with doxycycline 100 mg twice daily for 21 days.

However, 18 hours after starting doxycycline, the patient’s condition deteriorated and he became acutely ill. He complained of overwhelming fatigue, fever, chills, nausea, vomiting, and pain in his knees and ankles. He was encouraged to go to the emergency department, and on examination, significant scleral icterus was noted. Laboratory results revealed a decreased hemoglobin of 8.8 g/dL (reference normal, 13.0-17.7 g/dL), decreased platelet count of 74 × 10E3/uL (reference normal, 150-450 × 10E3/uL), elevated total bilirubin of 3.4 mg/dL (reference normal, 0.0-1.2 mg/dL), elevated direct bilirubin of 2.2 mg/dL (reference normal, 0.0-0.3 mg/dL), and elevated lactate dehydrogenase of 764 U/L (reference normal, <240 U/L). Lyme antibodies were now positive. As the laboratory findings were consistent with hemolytic anemia, acute Lyme disease, and constitutional symptoms, a blood smear was performed. His blood smear showed 8% parasitemia, with *Babesia microti* identified by rapid Giemsa stain. The patient was subsequently treated with doxycycline 100 mg twice daily, atovaquone 750 mg daily, and azithromycin 500 mg daily for 21 days, with a consequent resolution of his presenting symptoms and laboratory abnormalities.

## Discussion

Lyme disease is a prevalent zoonosis transmitted by the spirochete *Borrelia burgdorferi*, with various clinical manifestations at distinct stages of infection.^3^ The first stage of acute Lyme disease is characterized by erythema migrans at the site of the tick bite, although a history of a recent tick bite may be absent.^3^ This characteristic rash is present in up to 70% to 80% of infected patients, and often serves as the diagnostic clue, as positive serology can be delayed for up to several weeks after initial exposure to become detectable and histopathology is nonspecific, evidenced by this patient.^4^^,^^5^ Treatment should be empirically initiated in cases with high suspicion of Lyme after phlebotomy.

The JHR is a transient though potentially life-threatening phenomenon that develops within 24 hours after initiating antibiotic therapy for spirochetal infections.^1^ Although the reaction is most commonly associated with syphilis, it can occur on rare occasion after treating Lyme disease, as *Borrelia burgdorferi* is also a spirochete.^6^ The JHR has been reported to occur in 7% to 30% of treated Lyme disease cases.^6^ The exact pathomechanism has not been elucidated, although the release of endotoxin-like materials and elevated levels of inflammatory cytokines from the lysis of spirochetal organisms are thought to play a role.^7^ Clinically, JHR presents as an acute exacerbation of cutaneous symptoms and the abrupt onset of systemic symptoms, including fever, tachycardia, headache, nausea, and myalgia.^1^^,^^7^ This typically resolves without intervention and antibiotic therapy can be continued, although severe cases may require hospitalization.

Lyme disease is the most frequently transmitted pathogen by the *Ixodes* tick, although other pathogens, including anaplasmosis and babesiosis, are carried by the same tick.^2^ Rarely, coinfection of babesiosis with Lyme disease can occur.^8^ In a study of 52 patients with erythema migrans, evidence of coinfection with Babesia was detected in 4 (7.7%) of patients.^9^ Babesiosis presents with nonspecific constitutional symptoms, including fever, malaise, myalgia, or headache, occurring after parasite-mediated hemolysis of erythrocytes. Laboratory findings reveal hemolytic anemia, leukopenia, and thrombocytopenia.^10^ Early identification and treatment of babesiosis is critical to prevent potential complications of acute respiratory, renal, and congestive heart failures and disseminated intravascular coagulation.^8^

This case was remarkable in that not only did the JHR occur in association with Lyme disease, but also coinfection with babesiosis was subsequently identified. Although the symptoms of acute hemolytic anemia can occur with either the JHR or babesiosis, the timeframe of initiation of systemic symptoms after initiation of antibiotic treatment and exacerbation of a rash is characteristic of the JHR. To our knowledge, this is the first reported case of both the JHR and coinfection with Babesia presenting with Lyme disease. Eighteen hours after the first dose of doxycycline, the patient developed fatigue, fever, chills, nausea, and vomiting symptoms consistent with the JHR. Laboratory findings of hemolytic anemia and thrombocytopenia prompted evaluation for coinfection with Babesia. This case highlights the importance of maintaining high clinical suspicion for coinfection with additional tick-borne diseases when patients with Lyme disease acutely deteriorate or fail to respond to conventional therapy. Dermatologists should be cognizant of the potential of the JHR to occur in patients treated for Lyme disease in addition to its classic association with syphilis.

## Conflicts of interest

None disclosed.
